# Understanding the Tumor Microenvironment and Therapy Resistance in Head and Neck Squamous Cell Carcinoma

**DOI:** 10.3390/cells15010044

**Published:** 2025-12-25

**Authors:** Abhinav Bagchi, Ratna B. Ray

**Affiliations:** Department of Pathology, Saint Louis University, 1100 South Grand Boulevard, St. Louis, MO 63104, USA; abhinav.bagchi@health.slu.edu

**Keywords:** head and neck cancer, tumor microenvironment, therapeutic strategy

## Abstract

Head and neck squamous cell carcinoma (HNSCC) is a group of cancers arising in the oropharyngeal and laryngeal regions. Lifestyle choices such as smoking, tobacco chewing, alcohol consumption, and human papillomavirus (HPV) infection are key risk factors. Therapeutic options include surgical resection, chemoradiotherapy, EGFR-targeting therapy, and immunotherapy. However, the treatments are limited by drug resistance, relapses, and poor response to immunotherapy, especially in advanced diseases. The difference in tissue types and HPV infection status may lead to significant variations in their tumor microenvironment (TME). The heterogeneity contributes to poor treatment response and the development of therapeutic resistance. Therefore, it is critical to have a deeper understanding of the complexities and heterogeneity in TME and its role in treatment resistance. In this review, we focused on tumor heterogeneity and the role of cancer and non-cancer cells in therapeutic resistance. We discussed the studies on human HNSCC, especially HPV-negative, and presented the diversity in the tumor microenvironment and treatment response. Furthermore, we address the existing and experimental therapeutics that target therapy resistance and may lead to a better understanding of the disease and improve therapeutic outcomes.

## Simple Summary

Head and neck squamous cell carcinoma (HNSCC) is one of the most common cancers worldwide and is associated with tobacco use, alcohol consumption, and EBV or HPV infection. The treatment of HNSCC is improved with early detection by radiation and/or surgery, and immunotherapy. Unfortunately, many patients do not respond or develop resistance to these therapies. Here, we discuss the role of the tumor microenvironment and how cancer cells protect themselves by modulating key pathways, which may help in the development of novel therapeutics.

## 1. Introduction

HNSCC is a group of cancers arising in the oral cavity, pharynx, larynx, paranasal sinuses, nasal cavity, and salivary glands [1]. They originate in the squamous cells of the mucosa lining and often spread locally and to the surrounding lymph nodes. A recent study estimated almost 60,000 cases in 2025 and approximately 13,000 deaths in the United States [2]. In addition, GLOBACON estimates 890,000 cases and 450,000 deaths per year, making it seventh most common cancer [3]. Risk factors include alcohol and tobacco use, human papillomavirus (HPV) infection, Epstein–Barr virus (EBV) infection, tobacco chewing betel leaves (paan), exposure to construction materials like asbestos, metal, ceramic, wood dust, formaldehyde, and radiation [3]. Furthermore, genetic disorders like Fanconi anemia are also associated with an increased risk of developing HNSCC [4]. Two major classifications of HNSCC are based on HPV infection status [5]. HPV-positive cancers primarily arise in lingual and palatine tonsils and are associated with a better prognosis. HPV-negative cancers occur in non-oropharyngeal sites. At the molecular level, *TP53* inactivation or somatic mutation is a hallmark of HNSCC [6]. Furthermore, loss-of-function mutations in *CDKN2A*, *PTEN*, and NOTCH genes and gain-of-function mutations in *HRAS* and *CCND1* also contribute to the malignant transformation of the cells. Such mutations often lead to the activation of major pathways such as the Ras/MAPK and PI3K-mTOR that drive proliferation [7].

The standard of care for HNSCC includes surgical resection, radiotherapy, and platinum-based chemotherapy. Targeted therapies such as EGFR inhibitors are used when patients cannot take chemotherapy. For Recurrent or Metastatic HNSCC, anti-PD1 antibody (pembrolizumab) is a standard first-line therapy, often with platinum-based chemotherapy. However, only approximately 15% to 20% of these patients achieve durable responses [8]. The treatment response in HNSCC patients largely depends on the HPV infection status. HPV-positive patients respond better to treatments like immunotherapy than HPV-negative patients. HPV infection alters the tumor microenvironment by upregulating immune infiltration and antigen presentation, and is associated with better prognosis [9,10]. Therefore, effective treatments are necessary, especially in HPV-negative HNSCC patients.

The development of therapeutic resistance in HNSCC is a complex, multi-step process that warrants a deeper understanding to improve treatment outcomes. In this review, we summarize the molecular basis of therapy resistance with a special focus on the role of the tumor microenvironment in HPV-negative patients. This will help deepen our understanding of tumor heterogeneity and may help overcome therapeutic resistance and improve treatment efficacy.

## 2. Tumor Microenvironment and Tumor Heterogeneity in HNSCC

HNSCC microenvironment consists of a diverse tissue composition that contributes to the tumor structure. The tumor stroma in HNSCC includes neoplastic cancer cells and non-neoplastic stromal cells such as immune cells, fibroblasts, mesenchymal cells, endothelial cells, and nerves [11]. Stomal cells comprise the majority of the TME and interact with cancer cells to modulate tumor progression. A whole-exome sequencing (WES) study from 2015 analyzed bulk tumor DNA from 300 HNSCC patient samples for intra-tumor heterogeneity [12]. They developed a method called mutant-allele tumor heterogeneity (MATH) that was able to predict the overall survival of patients. The values for individual tumors were calculated using tumor-specific mutations in a genomic locus. A higher MATH value was associated with lower survival and vice versa [12]. Here, we will discuss how cancer and non-cancer cells impact TME and contribute to tumorigenicity.

### 2.1. Cancer Stem Cells

Cancer stem cells (CSCs) are pluripotent cells that drive the initiation, maintenance, and progression of tumors. They portray features of embryonic stem cells that retain or regain the potential of unlimited cell division and differentiation. The origin of CSCs is an area of active research, but it is widely believed that they originate from normal stem cells when they undergo several events, predominantly starting with alterations in the genome [13]. CSC populations are extremely rare in the TME, but they play a seminal role in tumor development [14]. One of the widely used CSC markers is octamer-binding transcription factor 4 (OCT4), which is primarily expressed in embryonic stem cells. In HNSCC patient samples, OCT4 was found to be differentially expressed and imparts stem-like properties to differentiated HNSCC cells. Furthermore, disruption of OCT4 attenuated the stem-like properties in these cells and limited tumor formation in vivo [15]. Nanog, CD44, CD133, and SOX2 expression were also higher in high-grade HNSCC samples compared to the low-grade tumors. The overexpression of these genes is associated with poor survival in HNSCC patients [16]. The CSC population contains cells expressing CD44 at the cell surface and BMI1 in the nucleus and possesses an extraordinary tumorigenic capacity. However, the abundance of CD44 expression in head and neck tissues made it difficult to use as a marker [17]. Interestingly, CD44 with c-met expression has been demonstrated as a very strong prognostic marker in a cohort of 105 patients [18]. High CD44 with c-met expression is associated with extremely poor clinical outcomes. Together, these studies lay a strong foundation to identify and study CSCs, especially using modern techniques such as spatial transcriptomics to delineate their role in the tumor microenvironment.

### 2.2. Immune Cells

The abundance and composition of immune cells in the TME have become a critical predictor of response to therapies, especially immunotherapy. The immune milieu is highly dynamic and varies significantly based on the tumor site, disease progression, and patient age, contributing to heterogeneity. Anti-tumor immunity is driven by immune cell-mediated cytotoxicity and antigen presentation. Cancer cells are usually flagged by our body to be targeted, but the tumors use a phenomenon called immunoediting to escape the surveillance [19]. Furthermore, cancers recruit immunosuppressive cells to limit the cytotoxicity from effector immune cells. Such modulations in the TME lead to dampened and dysfunctional immune responses. Hence, it is essential to understand the immune milieu and its crosstalk that leads to immune evasion to improve strategies for immunotherapy.

#### 2.2.1. Tumor-Infiltrating Lymphocytes

Higher tumor-infiltrating lymphocyte levels in HNSCC patients, particularly CD4 and CD8 T-cells, are associated with improved survival, and establishing them as a prognostic factor. Chemoradiation patients with high CD4 and CD8 presented a dramatic improvement in overall survival in a study involving 513 patients [20]. In the same study, high FoxP3 expression was also associated with improved overall survival in all patients, especially in chemoradiation patients. Another independent study on HNSCC patient samples presented improved overall survival associated with high cytoplasmic FoxP3 expression [21]. The authors reported FoxP3^+^ Treg as an independent prognostic factor in these samples, in combination with tumor stage and histological grade. This is in contrast with the general association of FoxP3^+^ regulatory T-cells (Tregs) with immune evasion and cancer progression in HNSCC. High FoxP3 transcript and protein levels in tongue squamous cell carcinoma patient samples have also been associated with poor survival [22]. However, the abundance of T-regulatory cells (Tregs) depends on the presence of lymphoid tissue around the tumor, and the role of FoxP3 Tregs in immune suppression may depend on the secretion of IL-12 and TGF-β in the tissues [23]. Interestingly, the subcellular localization of FoxP3 in Tregs may be vital in tumor progression. Immunofluorescence analysis of 49 OSCC patient samples revealed that subcellular localization of FoxP3 is essential to determine the clinical outcome, with cytoplasmic FoxP3 showing better survival than nuclear FoxP3 [24]. A nuclear vs. cytoplastic FoxP3 ratio can serve as a marker for recurrence in HNSCC. FoxP3, being a transcription factor, is generally located in the nucleus. However, the protein can also reside in the cytoplasm, which reduces its ability to regulate the Treg-associated gene expression and may affect its immunosuppressive functions [25]. A recent study on 354 clinical samples (including tumor and normal tissues) presented that high FoxP3 expression indeed associates with better survival in OSCC patients but such prediction requires several considerations [26]. FoxP3 expression regulates cell differentiation in cancer cells, and its role is not restricted to Tregs. The function of FoxP3 varies the isoform, subcellular localization, or the tissue origin, and may predict its clinical relevance. However, further research is needed to better understand the role of FoxP3 in HNSCC. The immune makeup within the HNSCC TME depends largely on the HPV infection status of the patients [27,28]. Partlova and colleagues analyzed 44 patient samples and demonstrated that HPV-positive tumors comprised elevated levels of CD8+ and CD4+ T-cells. CD8+ cells were isolated from the single cell suspension and stimulated in vitro, and it was demonstrated that CD8+ cells from HPV+ patients had greater potential to secrete IFNγ and IL-17. Furthermore, HPV-positive samples contained significantly higher myeloid dendritic cells (mDCs) and produced more chemokines when stimulated in vitro [27]. This demonstrates a strong immune phenotype in HNSCC patients based on HPV infection. Similarly, the analysis of 280 TCGA samples revealed increased CD8+ T-cell infiltration in addition to higher granzyme and perforin expression in HPV-positive samples [28]. The immune infiltration in HNSCC is inversely proportional to genomic instability, owing to increase immune surveillance in tumors containing chromosomally unstable cells. With the development of spatial-omics, recent studies are investigating the varying degrees of immune infiltration in HPV-negative HNSCC patients. A topological analysis of 53 HNSCC clinical samples revealed that intra-tumoral immune cells in HPV-negative HNSCC divulged varying infiltration of CD8^+^ T-cells. Based on the infiltration, the study classified tumors four categories—fully infiltrated, stroma-restricted, immune-excluded, and immune-desert. Fully infiltrated tumors expressed higher cytokine levels. The CD8+ T-cells were localized near tumor cells, whereas the B-cells remained isolated in exclusive niches [29]. In a separate study, tumor-associated B-cells were found to be more abundant in HPV-positive patients and were functionally involved in antibody-dependent immune responses [30]. Regulatory B-cells (Bregs) that produce adenosine (ADO) suppress T-cell function, regulated by Bruton’s tyrosine kinase (BTK) [31]. However, the abundance of ADO-producing Breg cells is less in the TME compared to the peripheral blood in patients. Further research is necessary to fully determine the role of Bregs in tumor progression and the modulation of immune responses. Together, the observations suggest that HPV status may affect immune cell infiltration. This attribute is associated with better prognosis and enhanced response to treatment in HPV-positive patients than in HPV-negative patients [32]. A summary with differences in immune composition in HPV-positive vs. HPV-negative HNSCC is presented in Table 1.

#### 2.2.2. Tumor-Associated Macrophages

The TME of HNSCC is characterized as immunosuppressive, dominated by the presence of tumor-associated macrophages (TAMs) [32,36]. In oral squamous cell carcinoma (OSCC) patient samples, metastatic tumors had the highest percentage of CD68^+^ TAMs, followed by non-metastatic samples and the control. High-CD68 cells were associated with worse survival in these patients. Furthermore, the trend was similar for the expression of anti-inflammatory cytokines such as IL-10 and TGF-β [37]. Such a population of TAMs is described as the M2 type, which promotes tumor progression. A study on primary nasopharyngeal carcinoma (NPC) patient samples presented immune-cell-specific signatures using single-cell transcriptomics [38]. The authors used marker genes to compute the signatures and presented that higher scores for NK cells, dendritic cells, and macrophages were associated with better progression-free survival in these patients. Similarly, M2 polarization plays a key role in the malignant transformation of oral leukoplakia (OLP) and aids in tumor progression. OLPs without macrophage infiltration are less likely to undergo malignant transformation [39]. Macrophage polarization to M2-type is characterized by CD163 expression in oral squamous cell carcinoma, and the CD163/CD68 ratio can be used as a measure for M2 polarization [40]. A 2024 study on publicly available scRNA-seq data from 18 HNSCC samples constructed a prognostic signature with eight macrophage-related genes (MRGs) that could predict risk in HNSCC patients. Low-risk patients are more likely to respond better to immunotherapy, and vice versa [41]. This suggests that TAMs are crucial in HNSCC progression and can be used as a prognostic marker.

#### 2.2.3. Myeloid-Derived Suppressor Cells

Myeloid-derived suppressor cells (MDSCs) promote malignant progression in HNSCC. A study using 200 OSCC samples and 36 normal tissues demonstrated that MDSC infiltration was associated with poor prognosis [42]. MDSCs isolated from human tumors showed that the presence of tumor-associated MDSCs led to higher proliferation of OSCC cells in vitro. Additionally, they promoted the invasion and migration phenotype and upregulated EMT-related genes in OSCC cells. HNSCC patients also revealed higher MDSC levels in the blood, especially with gross tumors [43]. Similar observations were made in the tobacco-mimicking 4-NQO-induced oral cancer animal model, where a gradual increase in MDSCs was observed starting from hyperplasia to carcinoma. Furthermore, MDSCs impede T-cell proliferation in vitro, with a marked reduction in the presence of tumor cells. Targeting MDSCs with sunitinib improved T-cell-mediated anti-tumor immunity by promoting infiltration, reducing T-cell exhaustion, and upregulating PD-L1 expression, which sensitized the tumors to anti-PD1 therapy [44]. Hence, the successful targeting of MDSCs may help overcome resistance to immunotherapy in HNSCC.

#### 2.2.4. Natural Killer Cells

Natural killer (NK) cell research has been in the limelight after the discovery of their significant role in anti-tumor immune response and the development of CAR-NK and NK-cell-based immunotherapy [45]. A single-cell RNA-seq study using human HNSCC samples revealed two distinct states—CD49a^+^ and CD49a^−^ cells [46]. CD49a^+^ cells express granzyme and perforin and play an anti-tumor role, whereas CD49a^−^ cells express NR4A2 that promotes Treg differentiation, leading to tumor suppression. A clinical trial with Metformin in combination with chemoradiotherapy induced NK-cell mediated cytotoxicity in HNSCC patients. It promoted NK cell infiltration and perforin expression via the mTOR/pSTAT1 pathway [47]. The analysis of PBMCs from HNSCC patients revealed increased TIM-3- and NKG2A-expressing exhausted NK cells compared to high CD56^bright^ cells in healthy donors [48]. Furthermore, cetuximab, an anti-EGFR antibody, promotes IFN-γ secretion in peripheral NK cells but not in intra-tumoral NK cells. Targeting TIGIT, a co-inhibitory molecule, also improves NK cell response in vitro and in pre-clinical models. Combining allogenic NLK cells with cetuximab yielded promising anti-tumor effects in vitro and in the xenograft model [49]. A combination of chemoradiotherapy with cisplatin enhanced NK cell infiltration and cytotoxicity, and improved overall survival in HNSCC patients [50]. Furthermore, NK cells, in the presence of natural compound bitter melon, enhanced killing activity when co-cultured with HNSCC cells [51]. Bitter melon extract increased the expression of CD16, a marker for NK cell activation and granzyme B, in vitro. Additionally, Car-NK cells targeting EGFR demonstrated enhanced cytotoxicity and apoptosis induction in SCC cell lines and primary HNSCC tumor cells [52]. However, more research is needed to determine their effectiveness in animal models to determine their translational potential.

#### 2.2.5. Antigen Presentation

In HNSCC, HLA class I antigen and associated antigen-presenting genes, such as TAP, are downregulated in primary and metastatic lesions in 25 clinical samples [53]. Histopathologic studies of HNSCC patients have revealed a significant reduction in HLA class I, TAP1, and TAP2 expression in metastatic lesions. Furthermore, lower HLA class I expression was associated with worse disease-free survival. In many cases, the antigen-presenting machinery presents functional defects despite the regular expression of antigen-presenting genes [54]. HPV infection alters antigen presentation in the TME. The analysis of TCGA data from HPV-positive oral tumors revealed an upregulation of MHC I and associated antigen presentation genes compared to HPV-negative samples. This was in contrast with in vitro studies that presented a reduction in the expression of antigen presentation genes. Despite contrasting observations, the author argued that the increase in viral-mediated antigen presentation was possibly due to the in vivo context [55]. Similarly, MHC II expressions are also upregulated in HPV-positive HNSCC patients, including both α and β chains. Additionally, associated antigen-presenting genes such as cluster of differentiation 74 (CD74), HLA-DM, HLA-DO, and their transcriptional regulators were overexpressed as well [10]. In a contrasting study, HLA-A expression was found to be significantly higher in HPV-negative patients, leading to higher clonal expansion of T-cells [56]. However, all the patients have been recipients of chemotherapy, and the difference in the HLA expression was barely significant. Overall, HPV-positive patients exhibit better antigen diversity due to the additional presence of virus-derived antigens, leading to a “hot” TME [57].

### 2.3. Stromal Cells

The cancer nests are protected inside a desmoplastic environment that is maintained by non-cancerous cells, including cancer-associated fibroblasts (CAFs), mesenchymal stromal cells (MSCs), endothelial cells, and extracellular matrix [58]. Cancer-associated fibroblasts (CAFs) are essential in tumor growth, with a more prominent role in extracellular matrix production [59]. CAFs originate from regular fibroblasts upon activation, primarily through transforming growth factor-β (TGF-β) and stromal cell-derived factor-1 (SDF-1) from cancer cells, and comprise a significant portion of the TME [60,61]. CAFs are classified into myofibroblastic CAFs, inflammatory CAFs, and antigen-presenting CAFs [62]. In HNSCC, CAFs are predominantly myofibroblastic and are identified using alpha-smooth muscle actin (α-SMA) expression. CAFs have been reported to promote and suppress tumor progression, and such a contrasting nature is an attribute of the heterogeneity and tissue specificity, and the manner of their activation [63]. A study on 72 HNSCC patients revealed that *AKT3* modulates CAF activity in the TME and is associated with poor prognosis. Loss of *AKT3* in vitro resulted in a reduction in immunosuppressive activity, characterized by decreased CCL2 expression and the elevation of M1-like macrophage genes, such as *IL12B* and *NOS2*. Knockdown of *PIK3CA*, an upstream molecule of *AKT3*, drastically affected CAF viability [64]. Another study in OSCC patient samples demonstrated that *CXCL12* expression by inflammatory CAFs promoted the infiltration of M2 macrophages, most likely via the *CXCR4*-*CXCL12* axis [65]. This suggests that heterogeneous populations of CAFs alter TME and, accordingly, responses to therapies. In contrast, CAFs have also been reported to suppress stemness in cancer stem cells derived from oral tumors. CAFs from primary gingivobuccal oral tumors with low α-SMA expression reduced stemness in oral cancer stem cells through the upregulation of *BMP4*. These CAFs are referred to as “C1-type” and were characterized by low α-SMA and high *BMP4* levels. CAFs with high α-SMA were C2 type, which promoted stemness [66]. CAFs are critical in tumor progression, and their heterogeneity and mechanisms in HNSCC need further investigation.

**Figure 1:**
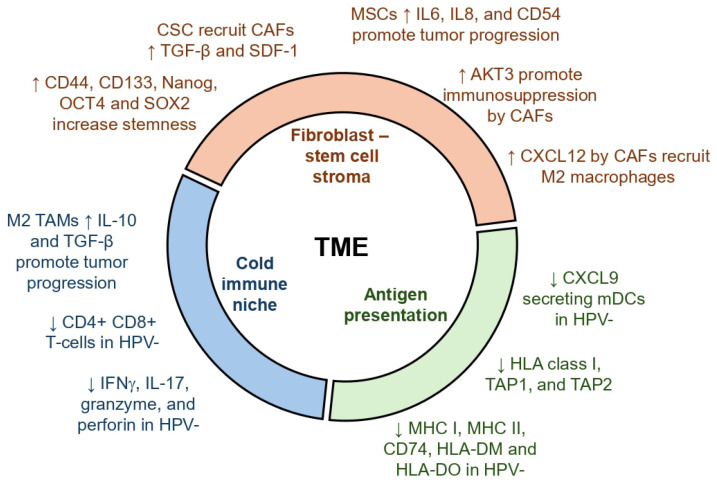
Role of various cells in the tumor microenvironment in HNSCC. Key events are broadly classified into three groups: stem cells and fibroblasts, immune cells, and antigen presentation. Up arrows indicate events upregulated and down arrows indicate events downregulated in the TME, which promote tumor progression.

MSCs play a key role in maintaining cancer stem cells [67]. Bone marrow-derived MSCs may serve as a source for CAFs in the TME, especially in gastric cancer [68]. However, the validity of the concept in head and neck cancers is poorly studied. Nonetheless, analysis of oropharyngeal squamous cell carcinoma patient samples revealed a greater abundance of MSCs compared to normal tissue [69]. Cell lines derived from tumors improved the migration of MSCs in vitro, driven by IL-6 and platelet-derived growth factor α (PDGFα), suggesting that a crosstalk between MSCs and cancer cells may promote desmoplasia in HNSCC. MSCs isolated from patients showed increased expression of IL6, IL8, and CD54 [70]. In a separate study, the upregulation of a connective tissue growth factor, CCN2, was identified in tongue squamous cell carcinoma samples. MSCs were the source of CCN2, and promoted cancer cell proliferation and cell migration [71]. Similarly, bone marrow-derived MSCs were reported to exhibit pro-tumorigenic properties on HNC cells in vitro, such as cell proliferation and migration, and inhibited cell death by activating the mTOR pathway [72]. In contrast, MSCs isolated from normal gingival tissue exerted anti-tumor effects in vivo and inhibited cancer cell growth through the JNK pathway in vitro [73]. The MSCs upregulated pro-apoptotic genes in cancer cells and downregulated cell cycle and proliferation genes. Hence, the role of MSCs in tumor growth may be tissue dependent in HNSCC. A summary of myriad roles of cells in the TME is depicted in Figure 1.

## 3. Therapeutics and Development of Resistance in Head and Neck Squamous Cell Carcinoma

Current therapeutics for HNSCC include the use of surgical interventions for early-stage cancers in addition to radiotherapy and chemotherapy. Advanced diseases are treated using targeted therapy and immunotherapy [74,75]. The most widely used chemotherapy in HNSCC is cisplatin, a platinum-based anti-cancer drug, combined with fluorouracil and taxanes like docetaxel and paclitaxel. However, a major limitation of chemotherapy is the development of resistance. Immunotherapies include nivolumab and pembrolizumab, which are anti-PD1 monoclonal antibodies that have demonstrated promising outcomes, especially in patients with a higher PD-L1 expression and a greater mutational burden [76,77,78]. Immunotherapy has been able to improve overall survival in HNSCC patients but the treatment is limited by severe adverse effects and a low response rate [75,79]. Epidermal growth factor receptor (EGFR) is a tyrosine kinase present on the plasma membrane. The EGFR signaling pathway is fundamental for cell proliferation and is one of the most widely targeted signaling pathways. EGFR-targeting chimeric monoclonal antibody cetuximab has been approved by the FDA to treat HNSCC. Cetuximab sensitizes tumors to chemoradiation therapy and is used for locally advanced HNSCCs. However, it induces serious skin toxicity which limits its use in the clinic [79,80]. Gefitinib, a small molecule EGFR inhibitor, in combination with IFN-α, delayed tumor growth in a HNSCC xenograft model. Gefitinib suppressed EGFR activation and induced a pro-apoptotic effects of IFN-α [81].

Novel strategies under investigation include experimental therapeutics or FDA approved drugs which are being repurposed, with a special focus on combination therapies. EGFR inhibitor erlotinib with anti-VEGF antibody bevacizumab, in combination with radiation therapy, showed remarkable tumor inhibition in a xenograft model [82]. Conversely, the therapy failed to confer clinical benefits due to resistance via upregulation of nerve growth factor (NGF)-TrkA axis [83]. Recently, a pan-HER (HER is a part of the EGFR family) inhibitor, dacomitinib, and PI3K/mTOR inhibitor, gedatolisib, successfully attenuated tumor growth in combination with radiation therapy in xenograft models. Interestingly, they did not portray additional inhibition when used simultaneously, which suggested a complicated biology of dual inhibition of EGFR and PI3K/mTOR pathways [84]. Like bevacizumab, a dual VEGF-2 and FGFR1 inhibitor, lenvatinib targets angiogenesis and portrays robust anti-tumor properties in nasopharyngeal carcinoma [85,86]. Additional VEGF inhibitors, such as linifanib, accentuated radiosensitivity in radio-resistant HNSCC cells via STAT3 inhibition and the induction of apoptosis [87]. Fibroblast growth factor receptor (FGFR) inhibitor AZD4547 could block the phosphorylation of MAPK and promote radiosensitivity in HNSCC cell lines. Furthermore, AZD4547 arrested tumor growth in xenograft and PDX models [88]. Other such molecules have been developed to target c-MET [89,90], MEK [91,92], JAK/STAT [93,94], and CDK4/6 [95] in HNSCC, but the efficacy was limited by therapeutic resistance. Furthermore, the oral administration of bitter melon extract (BME) reduced tumor growth in an oral cancer xenograft model by disrupting cell cycle genes [96]. A representative list of recent clinical trials targeting TME is presented in Table 2.

*Strategies Targeting Therapeutic Resistance*

Cancer is more than a mass of proliferating cells and is a complex heterogeneous tissue that includes cancer and non-cancer cells in its microenvironment. The tumor microenvironment supports tumor growth and aids in stress mitigation upon treatment [97]. Broadly, cancers exhibit two types of stress response—primary and adaptive. Primary stress responses include phenomena such as the Warburg effect, where cancer cells utilize glycolysis to generate energy more efficiently, even in the presence of oxygen, and the stress eliciting the primary response is predominantly innate and is generated due to the natural course of tumor growth. However, an adaptive response develops over time in the presence of external stress, such as therapies. Both tumor intrinsic (genetic) and tumor extrinsic (non-genetic) pathways drive the development of acquired resistance [98]. Hence, the mechanisms arise from dynamic evolutionary processes where the genotype (mutations) leads to epigenetic plasticity. As the tumor grows, it gathers mutations and becomes genetically heterogeneous. The heterogeneity results in distinct molecular signatures, leading to varying sensitivity to therapies [99].

In HNSCC, key pathways that contribute to the development of resistance are epigenetic modulations, the deregulation in DNA repair pathways, the evasion of programmed cell death, and the re-wiring of major cell signaling and metabolic pathways [100]. Such changes promote resistance to radiation therapies, chemotherapies, targeted therapies, and immunotherapies. Major pathways that contribute to resistance are histone acetylation and epigenetics, defects in DNA repair, evasion of cell death, and immunosuppression [100,101]. Epigenetic alterations that promote resistance are DNA methylation, histone modifications, and miRNA alterations [102]. For example, DNA methylation is profoundly associated with resistance to radiation therapy. A study using two HNSCC cell lines—one radiation sensitive and one radiation resistant—showed that radiation-resistant cells had increased DNA methylation and differentially expressed 84 related genes between the cells [103].

Cisplatin is one of the most widely used chemotherapies, but many patients develop resistance, leading to relapse. The resistance is attributed to increased DNA repair, decreased cellular uptake of cisplatin, and cytosolic inactivation of cisplatin [104]. In HNSCC, high levels of ERCC1, an endonuclease, are associated with the increased nucleotide excision repair (NER) pathway. NER promotes cisplatin resistance by upregulating double-strand break repair induced by radiation. A clinical study revealed that ERCC1 can be used as a predictor for treatment response in HNSCC patients [104,105,106]. Furthermore, accumulation of miR-21 upregulates the expression of PDCD4 which promotes resistance to cisplatin [107]. Similarly, miR-23a inhibits cisplatin-induced apoptosis in tongue squamous cell carcinoma cells via the upregulation of JNK-dependent Twist expression [108]. Wild-type p53 and high levels of Bcl-xl are also associated with cisplatin resistance. Bcl-xl inhibits programmed cell death and promotes cisplatin resistance. High Bcl-xl is associated with poor outcomes in HNSCC [109]. Targeting Bcl-xl and other BCL2 family proteins using small molecules has demonstrated anti-tumor properties in HNSCC, especially in association with MCL-1 blockers [110]. CSCs promote chemoresistance and tumor progression, attributed to their unlimited self-renewal capability and rapid proliferative potential. Small molecules such as ABT-737 have been used to target CSCs in HNSCC. ABT-737 is a BH3-mimetic molecule that inhibits anti-apoptotic BCL-2 proteins to induce programmed cell death [111]. This molecule specifically targets cancer cells and can be used as an adjuvant to radiotherapy.

EGFR-targeting agent cetuximab is the only EGFR-targeted therapy approved by the FDA. Studies using cetuximab resistance models revealed a nuclear translocation of EGFR in resistance cells, in addition to the upregulation of MAPK, Ras, and mTOR/AKT signaling. Furthermore, the upregulation of EMT markers such as CD44 was observed as well [112]. Interestingly, whole-exome and RNA sequencing of HNSCC biopsies pre- and post-cetuximab treatment identified a mutation in the extracellular domain of EGFR. The mutation disrupted cetuximab binding to EGFR, suggesting a novel mechanism of cetuximab resistance and potentially a general mechanism of acquired resistance against targeted therapies [113]. In addition, metabolic reprogramming also contributes to the anti-EGFR therapy resistance in HNSCC. Cetuximab-resistant cells display a reprogramming of lipid metabolism mediated by peroxisome proliferator-activated receptor alpha (PPARα). Increased fatty acid uptake and stearoyl-CoA desaturase activity made the cells resistant to lipotoxicity, mediated by PPARα [114]. Momordicine-I (M-I) is a bioactive, stable, and non-toxic metabolite present in bitter melon [115]. M-I treatment suppresses HNSCC growth in vitro and in vivo by altering lipid metabolism and the induction of autophagy, leading to a reduction in tumor volume in animal models [116]. Resistance to EGFR therapies is attributed to reduced EGFR expression and the activation of compensatory pathways upon EGFR blocking [117]. Combining cetuximab with M-I may yield better outcome in HNSCC models and needs to be validated. Metabolic reprogramming contributes to the resistance of targeted therapies, and disrupting those pathways may provide a therapeutic window in HNSCC.

**Figure 2:**
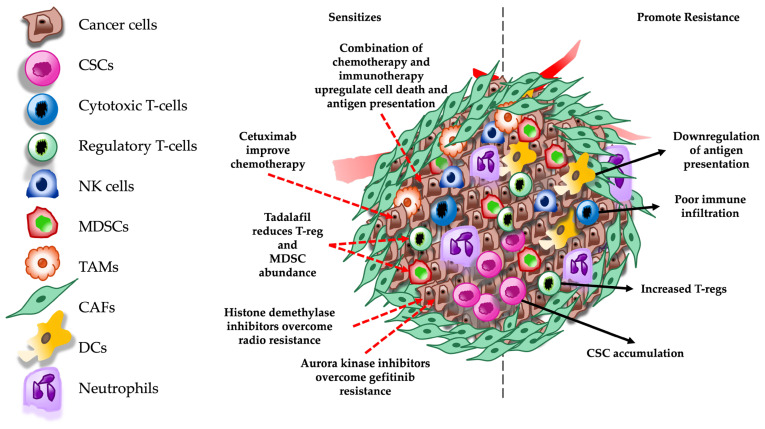
Schematic representation of molecular mechanisms involved in therapeutic resistance and pathways targeted to overcome resistance in the TME. Red dashed arrows indicate therapeutic targets and black dashed arrows indicate mechanisms that promote therapeutic resistance in the TME. CSCs = cancer stem cells; NK = natural killer; MDSCs = myeloid derived suppressor cells; TAMs = tumor-associated macrophages; CAFs = cancer associated fibroblasts; and DCs = dendritic cells.

Resistance to immunotherapy is an area of active research in HNSCC. As mentioned earlier, HPV infection status plays a key role in defining the immune signature in the TME of HNSCC. HPV-positive patients generally present better tumor infiltration of immune cells, which is associated with better outcomes. However, HPV-negative tumors display an immunosuppressive environment characterized by poor tumor infiltration and the inadequate antigen presentation associated with lower mutational burden. Furthermore, TME also consists of higher immunosuppressive cells, such as T-regs and MDSCs [101]. Tumor galectin-1 (Gal-1) levels were inversely related to immune checkpoint inhibitor (ICI) treatment in HNSCC patients [118]. Gal-1 promoted immunosuppression by inhibiting T-cell infiltration. Likewise, the inhibition of Gal-1 resensitized the tumors to immunotherapy and significantly reduced tumor growth when combined with radiation therapy. To overcome the lack of antigen presentation, a combination of cisplatin and anti-PD1 immunotherapy was used under the rationale that cisplatin may enhance antigen presentation in the TME by inducing immunogenic cell death [119]. This study showed that sublethal doses of cisplatin in combination with anti-PD1 delayed tumor growth in animal models. Together, this suggests that the induction of immunogenic cell death and inhibiting molecules like Gal-1 may improve immunotherapy in HNSCC. M-I treatment displays robust anti-tumor effects in mouse HNSCC xenograft tumors by affecting the TAM population [120]. M-I treatment promotes a switch from M2 to M1 phenotype, as demonstrated by an inhibition of *Arg1* expression. Furthermore, the study presented a reduction in the expression of myeloid cell differentiation factor *Sfln4* and neutrophil chemoattractant *Cxcl3*. M-I treatment also downregulated the expression of *PD1*, *PD-L1*, and *FoxP3* in the tumor, suggesting a strong candidate for immunotherapy in HNSCC [120]. Some key mechanisms promoting therapeutic resistance and strategies used to overcome resistance are listed in Table 3. Furthermore, a schematic representation of these mechanisms is depicted in Figure 2. Overall, poor immune infiltration with increased immune suppressive mechanisms such as an increase in T-reg and MDSC population and decrease in antigen presentation in addition to the accumulation of CSCs promote resistance to chemotherapy and immunotherapy. However, targeting these mechanisms using a combination of chemotherapy, immunotherapy, and/or targeted therapy such as cetuximab, tadalafil, and aurora kinase inhibitors have exhibited significant improvements in therapeutic outcomes.

## 4. Emerging Areas in HNSCC TME Research

The development of spatial technology and the integration of multi-omics are state-of-the-art methods that are providing novel insights that may help us improve existing therapies, especially immunotherapy [156,157]. Single-cell RNA-seq helped us study the gene expression in a specific cell type within the TME and adding spatial knowledge allowed us to visualize the proximity of such signatures. For example, a recent study using surgery samples from HNSCC patients revealed that CAFs overexpressing MHC-I in the stroma limits CD8+ cell infiltration by enriching galectin-9 production, which is the ligand for Tim-3 in CD8+ cells. This engages the CD8+ T-cells in the tumor stroma and inhibits immune infiltration into the tumor nest [158]. Similarly, a study using four male, HPV-negative HNSCC patient samples revealed that immunologically active tumors demonstrate a proximity of regions that are homogenous for immune cells, such as CD8+ T-cells. Furthermore, colocalization of PD1 high cytotoxic T-cells with cytotoxic T-cells demonstrate greater exhaustion, despite having high immune infiltration, leading to poor anti-tumor immune response [33].

The oral, gut, or tumor microbiome have a significant impact on the response to immunotherapy in HNSCC. A large study including 2724 patients demonstrated a significant decrease in the efficacy immunotherapy pembrolizumab when administered with antibiotics. The authors hypothesized that the difference was most likely due to a change in gut microbiome and warranted detailed microbiome study in a clinical setting [159]. Another study on early stage HNSCC patients treated with immunotherapy durvalumab revealed no change in the oral microbiome. The differences observed were owed to the presence of HPV infection [160]. The small sample size and the duration of the study may have different outcomes. However, the endogenous microbiome may play little role in the efficacy of immunotherapy, but the use of antibiotics and disrupting natural microbiome alter the response. Moreover, additional checkpoint blockers must be examined to achieve a more holistic understanding.

## 5. Future Perspectives

HNSCC is composed of a heterogeneous group of cancers with diverse anatomical locations which have distinct tumor microenvironments. Despite recent advances, HNSCC treatment is limited by the lack of effective targeted therapies, resistance to chemoradiation therapy, and the low response rate to immunotherapy, especially in HPV-negative patients. Therefore, targeting the tumor microenvironment to improve response and overcoming therapeutic resistance remain key challenges in head and neck cancers. A phase 3 clinical trial on patients with locally advanced HNSCC revealed that neoadjuvant and adjuvant administration of pembrolizumab in addition to chemoradiation therapy significantly improved event-free survival [161]. For patients unfit for chemotherapy, a combination of radiotherapy (RT) and pembrolizumab/cetuximab has been tested. However, RT + pembrolizumab did not improve tumor control in comparison with RT + cetuximab, but the RT + pembrolizumab combination appeared to be more tolerable [162].

In addition to PD-L1, which is most widely used biomarker, the identification of more novel biomarkers is necessary to predict the outcome of immunotherapy response. Secretome analysis of patient-derived HNSCC explants revealed transcriptome signatures that were defined as activation (Act) and infiltration (Inf) phenotypes [163]. The Act phenotype included expression of markers such as IFNγ, GZMH, and PD-L1, whereas the Inf phenotype included T-cell markers like CD4 and CD8. Both phenotypes were correlated with T-cell functionality and could predict survival and response to immunotherapy. Interestingly, the Inf phenotype was closely associated with HPV-positive patients, highlighting the importance of HPV status in treatment response. Nevertheless, a larger and more diverse cohort of patients may strengthen the observation and may be potentially used in the clinic. Recently, tertiary lymphoid structures (TLSs) are studied to predict immunotherapy outcomes. TLSs are generally described as an immune niche that replicates secondary lymphoid organs with CD20+ B-cells at the core surrounded by CD3+ T-cells, including CD4+ and CD8+ T-cells [164,165,166]. In 247 HPV-negative HNSCC, the presence of tertiary lymphoid structures (TLSs) correlated with better survival and an improved response to immunotherapy [167]. The immune stratification using transcriptomics and IHC data from patients provides a robust platform to develop immune subtypes and may serve as a promising strategy to improve immunotherapy, especially in HPV-negative patients. Furthermore, overexpressing TNFSF14 (also known as LIGHT) can improve vasculature and promote TLS formation in HNSCC-negative tumors via lymphotoxin β receptor (LTβR) signaling [168]. LIGHT induces chemokine production and boosts T-cell recruitment to tumors and helps overcome resistance to ICIs [169,170]. Additionally, BME and M-I treatment has exhibited robust anti-tumor effects in various HPV-negative HNSCC pre-clinical models, especially in immunotherapy resistant MOC2 tumors [120,171]. However, additional research is necessary to investigate the potency in combination with chemotherapy or ICIs, which may improve therapeutic outcomes in HPV-negative patients. Further, clinical studies will support the findings.

## Figures and Tables

**Table 1:** Differences in immune composition in HPV-positive and HPV-negative HNSCC.

TME Feature	HPV-Positive	HPV-Negative	Model	Reference
**T-cells (CD4+ and CD8+)**	High	Low	Clinical/TCGA	[27,28]
**Myeloid dendritic cells**	High	Low	Clinical	[27]
**Granzyme/Perforin expression**	High	Low	TCGA	[28]
**B-cells**	High	Low	Clinical	[30]
**MHC-I and MHC-II expression**	High	Low	TCGA	[10,33]
**Response to immunotherapy**	Good	Bad	Clinical	[34]
**Clinical outcome**	Good	Bad	Clinical	[35]

**Table 2:** Recent and ongoing clinical trials with strategies targeting TME in HNSCC.

TME-Targeted Strategies	Trial ID	Phase	Interventions	Status
Dual targeting of EGFR and TGF-β	NCT06788990	2/3	Ficerafusp alfa and Pembrolizumab	Recruiting
EGFR targeting cetuximab after immunotherapy	NCT04375384	2	Cetuximab	Recruiting
Anti-PD1 in combination with PARP inhibitor	NCT04681469	2	Dostarlimab and Niraparib	Recruiting
PDE-5 inhibitor to reprogram TME and boost immune cells in combination with ICB	NCT03993353	2	Tadalafil and Pembrolizumab	Active, not recruiting
Cytokine targeting IL-2 receptor, activating CD8+ T-cells and NK cells, in combination with ICB	NCT04144517	2	Nemvaleukin Alfa and Pembrolizumab	Complete, promising anti-tumor activity
Photoimmunotherapy (PIT) in combination with ICB or standard or care with ICB	NCT06699212	3	ASP-1929, Pembrolizumab and Chemotherapy	Recruiting
PI3Kγ inhibitor targeting immunosuppressive TAMs	NCT03795610	2	IPI-549	Completed

**Table 3:** Mechanisms of therapeutic resistance and strategies targeting resistance in HNSCC.

Type of Therapy	Effect on Therapeutic Resistance	Mechanisms	Evidence Level	Reference
**Radiation therapy**	**Promotes**	DNA methylation	A	[121,122,123]
		Upregulation in DNA double-strand break repair	A	[124,125]
		Upregulation in the PI3K/AKT/mTOR pathway	A	[126,127,128]
		Histone demethylase inhibitor GSK-J1sensitizes radioresistant HNSCC cells	A	[122]
		CDK4/6 inhibitor palbociclib radio sensitizes HPV-negative HNSCC	D	[129]
		Metformin radio sensitizes by inducing ROS production	A	[130]
**Chemotherapy**	**Promotes**	Histone modifications by NFkappaB	D	[131]
		Hypermethylation of the DNA repair gene	A	[102,132,133]
		Chromatin remodeling and CSC accumulation	A	[102,134,135]
		Differential expression of miRNAs	A	[136,137,138]
	**Sensitizes**	Epigallocatechin gallate induces apoptosis in cisplatin-resistant oral cancer cells	D	[139]
		HDAC inhibitors sensitize CSCs to cisplatin	B	[140,141]
		Drug delivery using nanoparticles	B	[142]
		Combination of cisplatin and cetuximab improves chemotherapy	A	[143,144,145]
**Targeted therapy**	**Promotes**	Hypoxia and EMT causes resistance to gefitinib	C	[146]
		Upregulation of fatty acid metabolism contributes to cetuximab resistance	C	[114]
		STAT3 upregulation promotes resistance to CDK4/6 inhibitors	C	[147]
		Increased Akt phosphorylation promotes Cetuximab resistance	D	[148]
	**Sensitizes**	Farnesyltransferase inhibitor tipifarnib enhances cetuximab efficacy	C	[149]
		Epiregulin inhibition improves cetuximab sensitivity by inducing ferroptosis	D	[150]
		Drug cocktails determined using patient-specific signaling signature (PaSSS) boost erlotinib therapy	C	[151]
		Targeting aurora kinase inhibits gefitinib-resistant HNSCC cells	B	[152,153]
		STAT3 inhibitor Stattic overcomes palbociclib resistance	C	[147]
		PPARα and FAO inhibitors overcome cetuximab resistance	C	[114]
		Combination with Akt inhibitor MK2206 overcomes cetuximab resistance	D	[148]
**Immunotherapy**	**Promotes**	HPV-negative patients have poor immune infiltration leading to an immunosuppressive TME	A	[101]
		Downregulation in antigen-presenting machinery	A	[54]
		Increased frequency of regulatory T-cells	A	[154]
	**Sensitizes**	Combination of chemotherapy and immunotherapy upregulate cell death and increase antigen presentation	A	[78]
		M-I monotherapy sensitizes non-responsive tumors in vivo	C	[120]
		Galectin-1 blockade improves anti-PD1 therapy	C	[118]
		Inhibition of PDE5 using tadalafil reduces MDSC and T-reg abundance	B	[155]

Evidence levels: A—Clinical evidence, B—clinical trials, C—pre-clinical animal studies, and D—in vitro or mechanistic evidence only.

## Data Availability

No new data were created or analyzed in this study.

## Abbreviations

AKT3	AKT Serine/Threonine Kinase 3
BCL2	B-Cell Lymphoma 2
BME	Bitter Melon Extract
CAF	Cancer Associated Fibroblast
CCN2	Cellular Communication Network Factor 2
CCND1	Cyclin D1
CD	Cluster of Differentiation
CDK	Cyclin-Dependent Kinase
CDKN2A	Cyclin-Dependent Kinase Inhibitor 2A
CSC	Cancer Stem Cell
CTLA4	Cytotoxic T-Lymphocyte-Associated Protein 4
CXCL9	C-X-C Motif Chemokine Ligand
DMBA	7,12-Dimethylbenz(A) Anthracene
EGFR	Epidermal Growth Factor Receptor
EMT	Epithelial-To-Mesenchymal Transition
FoxP3	Forkhead Box P3
GMDSC	Granulocytic Myeloid-Derived Suppressor Cell
HER	Human Epidermal Growth Factor Receptor
HLA	Human Leukocyte Antigen
HNSCC	Head and Neck Squamous Cell Carcinoma
HPV	Human Papillomavirus
HRAS	Harvey Rat Sarcoma Virus
IL	Interleukin
JAK/STAT	Janus Kinase/Signal Transducer and Activator of Transcription 3 Pathway
JNK	C-Jun N-Terminal Kinase
MAPK	Mitogen-Activated Protein Kinase
MATH	Mutant-Allele Tumor Heterogeneity
MCL	Myeloid Cell Leukemia
mDC	Myeloid Dendritic Cell
MDSC	Myeloid-Derived Suppressor Cell
MRG	Macrophage-Related Gene
MSC	Mesenchymal Stem Cell
mTOR	Mammalian Target of Rapamycin
NER	Nucleotide Excision Repair
NGF	Nerve Growth Factor
NOS2	Nitric Oxide Synthase 2
NOTCH	Neurogenic Locus Notch Homolog Protein
NPC	Nasopharyngeal Carcinoma
OCT4	Octamer-Binding Transcription Factor 4
OLP	Oral Leukoplakia
OSCC	Oral Squamous Cell Carcinoma
PD-L1	Programmed Cell Death Ligand 1
PD1	Programmed Cell Death Protein 1
PDCD4	Programmed Cell Death 4
PDGF	Platelet-Derived Growth Factor
PI3K	Phosphoinositide 3-Kinase
PIK3	Phosphatidylinositol-4,5-Bisphosphate 3-Kinase
PPAR	Peroxisome Proliferator-Activated Receptor
PTEN	Phosphatase and Tensin Homolog
SMA	Smooth Muscle Actin
SOX	SRY-Box Transcription Factor
T-Reg	Regulatory T-Cell
TAM	Tumor-Associated Macrophage
TAP	Transporter Associated with Antigen Processing
TGF	Transforming Growth Factor
TIL	Tumor-Infiltrating Lymphocyte
TME	Tumor Microenvironment
TRKa	Tropomyosin Receptor Kinase A
VEGF	Vascular Endothelial Growth Factor
WES	Whole-Exome Sequencing
